# Adipokines and Cardiometabolic Heart Failure with Preserved Ejection Fraction: A State-of-the-Art Review

**DOI:** 10.3390/diagnostics14232677

**Published:** 2024-11-27

**Authors:** Nikolaos Theodorakis, Magdalini Kreouzi, Christos Hitas, Dimitrios Anagnostou, Maria Nikolaou

**Affiliations:** 1 School of Medicine, National and Kapodistrian University of Athens, 75 Mikras Asias, 11527 Athens, Greece; nikolaostheodorakis1997@yahoo.com; 2 Department of Cardiology & Heart Failure Outpatient Clinic, Sismanogleio-Amalia Fleming General Hospital, 14 25is Martiou Str., 15127 Melissia, Greece; ch.chitas@flemig-hospital.gr (C.H.); jimdimitris100@gmail.com (D.A.); 3 Department of Internal Medicine, Sismanogleio-Amalia Fleming General Hospital, 14 25is Martiou Str., 15127 Melissia, Greece; kreouzi.m@live.unic.ac.cy

**Keywords:** adipokines, cardiometabolic medicine, obesity, heart failure with preserved ejection fraction, leptin, adiponectin, resistin, cardiology, endocrinology, metabolism

## Abstract

Background: Cardiometabolic heart failure with preserved ejection fraction (HFpEF) is largely driven by obesity-related factors, including adipokines and bioactive peptides primarily secreted by the adipose tissue, such as leptin, adiponectin, and resistin. These molecules link metabolic dysregulation to cardiovascular dysfunction, influencing HFpEF progression and patient outcomes Methods: A comprehensive literature search was conducted in PubMed up to 20 November 2024, using keywords and MeSH terms, such as “HFpEF”, “adipokines”, “leptin”, “adiponectin”, and “resistin”, yielding 723 results. Boolean operators refined the search, and reference lists of key studies were reviewed. After screening for duplicates and irrelevant studies, 103 articles were included, providing data on adipokines’ roles in HFpEF pathophysiology, biomarkers, and therapeutic implications. Results: Both preclinical and clinical studies have demonstrated that adipokines play a role in modulating cardiovascular function, thereby contributing to the development of cardiometabolic HFpEF. Leptin promotes myocardial hypertrophy, fibrosis, endothelial dysfunction, and inflammation, though contradictory evidence suggests potential cardioprotective roles in subgroups like obese African American women. Adiponectin generally offers protective effects but presents a paradox, where elevated levels may correlate with worse outcomes, which may reflect either a compensatory response to cardiac dysfunction or a maladaptive state characterized by adiponectin resistance. Resistin is associated with increased cardiovascular risk through pro-inflammatory and pro-fibrotic effects, though its role in HFpEF requires further clarification. Other adipokines, like retinol-binding protein 4 and omentin-1, have emerged as potential contributors. Despite growing insights, clinical translation remains limited, underscoring a significant gap between experimental evidence and therapeutic application. Conclusions: Future research should focus on targeted interventions that modulate adipokine pathways to potentially improve HFpEF outcomes. Innovative treatment strategies addressing underlying metabolic disturbances and adipokine dysregulation are essential for advancing the management of this challenging condition.

## 1. Introduction

The heart failure (HF) epidemic is the worldwide leading cause of hospitalizations, accounting for 2671 cases in Europe and 3793 in the United States per million population annually [1,2]. HF with preserved ejection fraction (HFpEF) is a condition with increasing prevalence, accounting for up to 65% of HF cases (1.4% of general population), which is further expected to increase as a product of population aging and the accumulation of cardiometabolic comorbidities [3]. Despite significant advancements, HFpEF remains a challenging condition with a complex pathophysiology that is not fully understood.

Obesity and metabolic syndrome have redefined the landscape of HFpEF, and emerging research highlights the role of adipokines—bioactive proteins secreted mainly by adipose tissue—as critical links between metabolic dysfunction and cardiovascular disease (CVD) [4]. Adipokines, including leptin, adiponectin, resistin, and others, are no longer viewed solely as markers of adiposity; they are active mediators in metabolic processes, inflammation, and cardiovascular remodeling. Their role in influencing myocardial stiffness, endothelial dysfunction, and systemic inflammation suggests a more direct involvement in HFpEF’s pathogenesis. Specifically, adipokines can exacerbate or attenuate cardiovascular dysfunction through their complex signaling networks, which become dysregulated in the context of metabolic diseases, such as obesity and type 2 diabetes mellitus (T2DM) [5].

While existing reviews on cardiometabolic HFpEF have extensively explored broad pathophysiological mechanisms, such as inflammation, fibrosis, endothelial dysfunction, and metabolic disturbances, the specific role of adipokines has not been comprehensively addressed. Moreover, prior reviews on adipokines have either taken a general approach or focused on their broader role in cardiovascular diseases, without specifically examining their impact on cardiometabolic HFpEF. This review aims to address this gap by focusing explicitly on adipokines and their involvement in the pathogenesis of cardiometabolic HFpEF. It delves into the current literature on the interactions between the major adipokines—leptin, adiponectin, and resistin—and cardiometabolic HFpEF, highlighting potential pathways through which they influence pathophysiology, identifying possible therapeutic targets, and emphasizing gaps and directions for future research.

## 2. Methods

This narrative review employed a comprehensive literature search to identify studies focusing on adipokines and their association with HFpEF. Using PubMed, we retrieved 723 articles published up to 20 November 2024. The search strategy included a variety of key words and MeSH terms, such as “HFpEF”, “heart failure”, “adipokines”, “leptin”, “adiponectin”, and “resistin”, as well as additional terms including “retinol-binding protein 4” (RBP-4), “apelin”, “visfatin”, “vaspin”, “omentin-1”, “chemerin”, “lipocalin-2”, “follistatin-like 1”, “isthmin-1”, and “dipeptidyl peptidase 4” (DPP-4). Boolean operators were used to combine these terms (e.g., adipokine OR leptin OR adiponectin OR resistin OR RBP-4, etc.). Boolean operators refined the search, and reference lists of key articles were reviewed to identify additional relevant studies. Initial screening removed duplicates and unrelated studies, resulting in 103 records included in the manuscript. Studies were included if they provided data on the role of adipokines in HFpEF pathophysiology, biomarkers, or therapeutic implications. This thorough approach ensured a comprehensive exploration of the role of adipokines in HFpEF.

## 3. HFpEF: Definition, Overview of Pathophysiology, and Biomarkers

The universal definition of HF encompasses the presence of signs/symptoms caused by cardiac structural/functional abnormalities that lead to cardiogenic pulmonary/systemic congestion and/or elevated natriuretic peptides. HF is further classified according to the left ventricular ejection fraction (LVEF) to HF with reduced ejection fraction (HFrEF–LVEF ≤ 40%), HF with mid-range ejection fraction (HFmrEF–LVEF 41–49%), and HFpEF (LVEF ≥ 50%) [6].

HFpEF and HFrEF are characterized by distinct pathophysiological mechanisms. HFrEF is marked by systolic dysfunction, often resulting from coronary artery disease (CAD), cardiomyopathies, long-standing severe valve diseases, or, less commonly, prolonged hypertensive heart disease. In contrast, HFpEF typically arises from a confluence of aging and various comorbidities, such as hypertension, obesity, T2DM, CAD, chronic kidney disease, chronic inflammatory conditions, and sleep disturbances, with a sedentary lifestyle further contributing to its development. Among these drivers, the combination of obesity and arterial hypertension are the most prominent contributors to the development of the HFpEF syndrome. This HFpEF phenotype has been defined as cardiometabolic HFpEF and initiates a series of interconnected pathophysiological and molecular mechanisms, including chronic inflammation, oxidative stress, and insulin resistance, that can lead to myocardial hypertrophy, fibrosis, apoptosis, endothelial dysfunction, and mitochondrial dysfunction. Together, these processes contribute to myocardial diastolic dysfunction—the hallmark of HFpEF—while also causing arterial stiffness and skeletal muscle dysfunction, both of which play significant roles in cardiometabolic HFpEF pathophysiology [4].

Skeletal muscle dysfunction is increasingly recognized as a key driver of cardiometabolic HFpEF. This dysfunction involves a shift from type I to type II muscle fibers, reducing the muscles’ capacity for aerobic metabolism and prompting earlier reliance on anaerobic glycolysis, thereby lowering the anaerobic threshold. This, in turn, leads to an exaggerated activation of the ergo-reflex, which stimulates the sympathetic nervous system, leading to tachypnea and increased venous return, further elevating left ventricular (LV) filling pressures [7]. Adding to this understanding, sex-specific associations between skeletal muscle mass and metabolic outcomes, such as incident T2DM, have been demonstrated. A study using the WATCH cohort found that a higher predicted skeletal muscle mass index (pSMI) was associated with a lower risk of developing T2DM, with important sex-specific differences. In women, a higher pSMI consistently correlated with reduced T2DM risk (hazard ratio [HR] per standard deviation increment: 0.79 [95% confidence interval [CI] 0.68–0.91]). In men, this association was nonlinear; those with pSMI below 8.1 benefited from increased muscle mass (HR: 0.58 [95% CI 0.40–0.84]); whereas, no significant association was observed for men with pSMI ≥ 8.1. These findings underscore the importance of skeletal muscle health in metabolic regulation, with implications for managing both T2DM and HFpEF [8].

Several biomarkers have been investigated to enhance the understanding of the pathophysiology, diagnosis, and prognosis of HFpEF. According to a recently published observational study of four large cohorts, notable biomarkers included natriuretic peptides (HR, 1.27; 95% CI, 1.16–1.40), the urinary albumin-to-creatinine ratio (HR, 1.33; 95% CI, 1.20–1.48), high-sensitivity troponins (HR, 1.11; 95% CI, 1.03–1.19), plasminogen activator inhibitor 1 (HR, 1.22; 95% CI, 1.03–1.45), and fibrinogen (HR, 1.12; 95% CI, 1.03–1.22) [9]. According to another study that investigated the association of protein biomarkers with incident HF among participants in the Framingham Heart Study, several biomarkers were associated with an increased risk of developing HFpEF. These included N-terminal pro b-type natriuretic peptide (NT-proBNP) (HR, 2.13; 95% CI, 1.52–2.99), growth differentiation factor-15 (HR, 1.67; 95% CI, 1.32–2.12), adrenomedullin (HR, 1.58; 95% CI, 1.23–2.04), uncarboxylated matrix Gla protein (HR, 1.55; 95% CI, 1.23–1.95), and C-reactive protein (CRP) (HR, 1.46; 95% CI, 1.17–1.83). These findings highlight the involvement of pathways related to cardiac stress, inflammation, and vascular stiffness in the development of HFpEF [10].

Furthermore, a study exploring the prognostic value of the aspartate aminotransferase to alanine aminotransferase (AST/ALT) ratio in patients with T2DM hospitalized for HF found that a higher AST/ALT ratio was associated with worse short-term outcomes. Patients in the highest quartile of the AST/ALT ratio had significantly increased risks of 30-day all-cause mortality (HR, 1.61; 95% CI, 1.18–2.19) and major systemic infection (HR, 1.28; 95% CI, 1.06–1.56), indicating the potential utility of this biomarker in risk stratification for this population [11].

These findings underscore that various novel biomarkers have been associated with HFpEF, while emerging research is also supporting the role of adipokines as potential biomarkers strongly associated with the pathophysiology of cardiometabolic HFpEF.

## 4. Adipokines in the Context of Cardiometabolic HFpEF

It was once widely believed that the impact of obesity on HFpEF was primarily due to physical or mechanical factors. However, in recent years, focus has been on the endocrine, metabolic, and cellular mechanisms underlying the cardiometabolic HFpEF phenotype. Emerging research suggests that cardiometabolic HFpEF may be driven by imbalances in adipokines, increased neprilysin activity, and enhanced mineralocorticoid signaling [12].

Adipose tissue in adults exists in four types, each with distinct locations and functions. White adipose tissue (WAT), the most abundant form, is primarily found subcutaneously (beneath the skin) and viscerally (around internal organs), serving as a long-term energy storage depot and playing a role in endocrine signaling. Brown adipose tissue (BAT) is located in areas like the neck, shoulders, and along the spine and is specialized in thermogenesis, generating heat through mitochondrial uncoupling. Beige adipose tissue emerges within white fat depots, particularly subcutaneous fat, in response to stimuli like cold exposure, and can shift between energy-storing and energy-burning functions. Lastly, pink adipose tissue, seen in mammary glands during pregnancy and lactation, supports milk production [13].

Adipose tissue acts as a significant endocrine organ. Adipokines are bioactive proteins secreted mainly by adipose tissue, which mediate a broad array of metabolic, immune, inflammatory, and cardiovascular processes. At this time, at least 615 adipokines have been discovered with ongoing research in the field [5]. According to a recently published review, adipokines can be classified according to site of production (WAT or BAT) and to their effects. Notable adipokines include, among others, leptin, adiponectin, resistin, apelin, omentin, visfatin, nesfatin, vaspin, chemerin, RBP-4, DPP-4, isthmin-1, and lipocalin-2. According to their effects, two major categories of classification for WAT adipokines include those with pro-inflammatory effects (e.g., leptin, resistin, visfatin, chemerin, DPP-4, and lipocalin-2) and those with anti-inflammatory effects (e.g., adiponectin, vaspin, apelin, omentin, isthmin-1, and nesfatin) [14].

This section delves into the specific roles and mechanisms through which some of the most notable adipokines—including leptin, adiponectin, and resistin—contribute to the pathophysiology of cardiometabolic HFpEF [8]. Understanding these connections is essential to developing targeted therapies that address both metabolic and cardiovascular components, with the aim of improving clinical outcomes for HFpEF patients.

### 4.1. Leptin

Leptin, a 16-kDa protein encoded by the obese (ob) gene, plays a central role in energy regulation by signaling satiety and promoting energy expenditure. It is primarily secreted from white adipose tissue with a diurnal pulsatile manner, with higher rates in the evening and early morning. In humans, circulating leptin levels positively correlate with body mass index (BMI), the percentage of body fat, total fat mass, and adipocyte size. Leptin receptors are encoded by the diabetes (db) gene, which undergoes alternative splicing of its pre-mRNA transcript to generate six distinct isoforms: Ob-Ra, Ob-Rb, Ob-Rc, Ob-Rd, Ob-Re, and Ob-Rf. Leptin receptors are expressed in multiple tissues, including the central nervous system (especially hypothalamus), cardiovascular system, adipose tissue, skeletal muscle, pancreas, liver, kidneys, and other tissues. The activation of these receptors triggers several intracellular signaling cascades, including the Janus kinase/signal transducers and activators of transcription (JAK/STAT), mitogen-activated protein kinase (MAPK), and phosphoinositide 3-kinase/protein kinase B (PI3K/Akt) pathways [15,16,17].

Leptin is primarily known for its ability to suppress appetite by binding to the Ob-Rb receptor in the arcuate nucleus of the hypothalamus and activating pro-opiomelanocortin (POMC) neurons, which release anorexigenic peptides, like alpha-melanocyte-stimulating hormone (α-MSH), to reduce food intake. Simultaneously, leptin inhibits neuropeptide-Y (NPY) and agouti-related peptide (AgRP) neurons, which are orexigenic and promote hunger, thereby further decreasing appetite [16].

Beyond its metabolic functions, leptin has profound cardiovascular effects mediated primarily through its long-form receptor (Ob-Rb), which is widely expressed in cardiovascular tissues, including the endothelium, myocardium, and vascular smooth muscle cells [15,16,17]. However, the effects of leptin on the cardiovascular system remain controversial.

Under acute conditions, leptin promotes a metabolic shift in cardiomyocytes toward glucose metabolism, which is more energy-efficient compared to fatty acid oxidation. This shift enhances energy production, while reducing myocardial oxygen consumption (MVO2), thereby improving cardiac efficiency. Additionally, leptin prevents myocardial lipid accumulation, protecting the heart from lipotoxicity and preserving metabolic flexibility. In leptin-deficient models, such as ob/ob or db/db mice, elevated triglyceride levels and lipid accumulation in the myocardium can promote lipotoxicity, impair cardiac contractility, and contribute to myocardial dysfunction. These acute effects of leptin may serve as a compensatory mechanism to mitigate cardiac stress during events like ischemia [16].

On the other hand, in humans, chronic hyperleptinemia has been linked to adverse effects on the heart and vasculature, highlighting its dual role in cardiovascular health. Two theories have been proposed to explain the adverse effects of chronic hyperleptinemia on the cardiovascular system. The first theory suggests that chronic leptin elevation leads to leptin resistance in all tissues, including the heart, resulting in the loss of leptin’s potential beneficial effects on cardiovascular health. The second and more widely supported theory proposes that chronic leptin elevation causes selective resistance in extracardiac tissues, while the cardiovascular system remains sensitive to leptin’s harmful effects. This concept of selective leptin resistance was first proposed in 2002 and further substantiated in 2013, particularly in relation to leptin’s effects on blood pressure [16,17,18,19].

Obesity leads to chronic leptin elevation, produced by epicardial adipose tissue or by dysfunctional subcutaneous or visceral adipose tissue. Furthermore, obesity is associated with elevated circulating levels of aldosterone, which can be secreted directly by adipocytes or released from the adrenal gland in response to leptin. This leptin-induced aldosterone release is further amplified in obesity due to the reduced anti-aldosterone effects of natriuretic peptides, a consequence of increased neprilysin activity, which results both from mature adipocytes and through kidney release triggered by sympathetic nerve activation. Furthermore, increased mineralocorticoid receptor activation, combined with a relative deficiency in natriuretic peptides, further drives adipose tissue expansion and dysfunction. The above three interconnected mechanisms—chronic hyperleptinemia, elevated aldosterone secretion, and decreased natriuretic peptide levels—lead to sodium retention and plasma volume expansion, sympathetic activation, inflammation, microvascular dysfunction, myocardial hypertrophy, and fibrosis. Additionally, the resulting dysfunctional cardiomyocytes can further secrete angiotensin II and leptin leading to a vicious cycle of chronic hyperleptinemia and myocardial dysfunction, contributing to cardiometabolic HFpEF [12,16,20,21]. The above mechanisms can potentially explain why finerenone, a non-steroidal mineralocorticoid receptor antagonist, has been proven to be effective in improving hard endpoints in patients with HFpEF, according to the FINE-ARTS trial [22].

Chronic hyperleptinemia has been shown to act centrally and increase sympathetic outflow, leading to elevations in blood pressure and heart rate. As a result of the above, chronic hyperleptinemia significantly increases myocardial afterload, which further increases LV filling pressures, decreases maximal cardiac output, and promotes the development of myocardial hypertrophy and fibrosis, contributing to the pathogenesis of hypertensive cardiopathy and HFpEF [23]. In this context, it is reasonable to consider that leptin may not directly cause myocardial hypertrophy and fibrosis, but rather, that these changes could represent a correlation. Hypertrophy observed in obese patients with elevated leptin levels might be attributed to increased blood pressure driven by both leptin-dependent and leptin-independent mechanisms, rather than a direct effect of leptin on the heart. Specifically, in preclinical models of obesity, leptin induces cardiomyocyte hypertrophy, while stimulating fibroblast proliferation and collagen synthesis mediated by the production of galectin-3, transforming growth factor-beta (TGF-β), and connective tissue growth factor production (CTGF) through oxidative stress increased by the activation of the mechanistic target of the rapamycin (mTOR) pathway [24]. A further mechanism by which chronic hyperleptinemia can promote ventricular hypertrophy and fibrosis is via increasing endothelin-1 (ET-1) production. Leptin stimulates ET-1 synthesis in endothelial cells through the activation of ObRb receptors. This process is further amplified by leptin-induced reactive oxygen species (ROS) generation, which upregulates ET-1 expression and exacerbates oxidative stress. The resulting ET-1 overproduction drives hypertrophy and fibrosis through direct pro-hypertrophic effects on cardiomyocytes and activation of cardiac fibroblasts, creating a feed-forward cycle of myocardial remodeling and dysfunction [16]. Furthermore, human studies provide additional evidence supporting the direct hypertrophic effects of chronic hyperleptinemia. In hypertensive, insulin-resistant men, fasting plasma leptin concentrations were found to correlate positively with myocardial wall thickness. Notably, this association remained significant even after adjusting for factors such as BMI, waist-to-hip ratio, and blood pressure, highlighting a potential independent role of leptin in influencing cardiac remodeling. Similarly, another study demonstrated a positive correlation between leptin levels and LV mass, even after accounting for BMI [18].

Beyond its effects on cardiac hypertrophy and fibrosis, leptin has also been implicated in the pathophysiology of HFpEF by its potential to affect myocardial lusitropy, with complex mechanisms implicating calcium handling. Leptin’s impact on myocardial lusitropy may involve disrupted calcium handling through altered sarcoplasmic reticulum Ca^2+^-ATPase (SERCA2a) and phospholamban (PLB) expression, key regulators of diastolic function. In HF models, hyperleptinemia correlates with downregulated SERCA2a and upregulated PLB, impairing calcium reuptake and relaxation. This dysfunction is linked to an activated ET-1 pathway and oxidative stress. CPU86017, an antiarrhythmic with antioxidant effects, has been shown to downregulate leptin and ET pathways, restoring SERCA2a and PLB expression and improving cardiac function, highlighting leptin as a potential therapeutic target in HFpEF [25].

Diabetic cardiomyopathy is characterized by abnormal myocardial structure and impaired function in the absence of other overt cardiovascular factors, such as CAD, valvular heart disease, hypertension, or dyslipidemia [26]. It progresses from asymptomatic LV diastolic dysfunction and hypertrophy to HFpEF and, later, HFrEF [26,27,28]. A recent study has linked elevated plasma leptin levels to LV hypertrophy, with higher leptin correlating with myocardial wall thickness independent from BMI and blood pressure [29]. As a result, chronic hyperleptinemia could be a key driver for the development of diabetic cardiomyopathy. Recent studies have highlighted the effectiveness of sodium–glucose cotransporter-2 inhibitor (SGLT2i) in addressing cardiac complications linked to obesity-related T2DM. Leptin plays a role in regulating SGLT2 expression in the kidneys, influenced by the sympathetic nervous system and the renin–angiotensin system. By counteracting the effects of leptin, SGLT2i helps reverse leptin-driven sodium and glucose retention in the kidneys and mitigates the associated inflammatory response in adipose tissue. This is achieved by reducing leptin secretion and its harmful paracrine actions on both cardiac and renal function [30]. Additionally, SGLT2i not only enhances cardiac performance and metabolism but also aids in weight loss through the reduction in visceral fat. They alleviate inflammation and insulin resistance induced by obesity by affecting M2 macrophage activity [31,32]. Importantly, these inhibitors have been shown to improve hard endpoints, including cardiovascular mortality and hospitalizations, in patients with HFpEF, irrespective of the presence of diabetes [33].

Chronic hyperleptinemia can lead to endothelial dysfunction by downregulating peroxisome proliferator-activated receptor (PPAR)-γ, a key protein involved in vasodilation through the promotion of nitric oxide (NO) production. Additionally, leptin drives the proliferation, migration, and calcification of vascular smooth muscle cells (VSMC), which play a significant role in the development of arterial stiffness and atherosclerosis. Moreover, leptin has pro-thrombotic properties, including the ability to stimulate platelet aggregation, while suppressing both fibrinolysis and coagulation, as further supported by mechanistic preclinical studies in mice with hyperleptinemia [34,35,36,37].

The study by Hubert et al. explored the impact of leptin, a hormone produced by adipose tissue, on vascular health in a cohort of 294 adolescents using high-resolution vascular ultrasound to assess brachial artery distensibility. The researchers found that higher leptin concentrations were significantly associated with reduced arterial distensibility, suggesting that leptin may impair vascular function independently from fat mass, blood pressure, or metabolic and inflammatory disturbances [38]. Furthermore, the study by Tsai et al. examined the relationship between serum leptin levels and arterial stiffness in 105 CAD patients using carotid–femoral pulse wave velocity as a marker. Results showed that patients with higher arterial stiffness were elevated leptin levels, which were significantly associated with increased arterial stiffness, independent from confounding factors, such as age, diabetes, and blood pressure. Additionally, leptin levels correlated with the number of stenotic coronary arteries, suggesting leptin’s potential role in the pathophysiology of arterial stiffness and CAD [39].

Chronic hyperleptinemia is also a significant pro-inflammatory mediator, stimulating the release of cytokines, like tumor necrosis factor-alpha (TNF-α) and interleukin (IL)-6, from macrophages and other immune cells, contributing to the development of chronic low-grade inflammation. This inflammatory response can exacerbate HFpEF by promoting endothelial dysfunction and driving further myocardial damage, illustrating leptin’s dual role in both metabolic and cardiovascular inflammation [40,41]. Pyrogallol-phloroglucinol-6,6-bieckol (PPB), derived from Ecklonia cava, was shown to significantly reduce inflammation and leptin resistance in diet-induced obese (DIO) and leptin-deficient (ob/ob) mice. PPB decreased pro-inflammatory markers, including TNF-α and IL-6, in the brain and adipose tissue, while suppressing macrophage activation and M1 polarization. It also attenuated endoplasmic reticulum stress, toll-like receptor 4 (TLR-4), and nuclear factor kappa B (NF-κB) expression in the brain, mechanisms that contribute to leptin resistance. By restoring leptin sensitivity, PPB effectively reduced food intake, body weight, and fat accumulation, highlighting its therapeutic potential in managing obesity-related inflammation and metabolic dysfunction [42].

In obesity, there is development of leptin resistance, where high leptin levels no longer effectively signal satiety, resulting in chronic hyperleptinemia. This resistance impairs the activation of the PI3K/AKT signaling pathway, which diminishes the recruitment of insulin receptor substrates 1 and 2 (IRS1 and IRS2), ultimately contributing to insulin resistance. Despite systemic resistance, cardiovascular tissues often retain sensitivity to leptin’s deleterious effects, a phenomenon called selective leptin resistance, which is particularly concerning in HFpEF. This allows elevated leptin to perpetuate pro-hypertrophic, pro-fibrotic, and pro-inflammatory actions on the heart and vasculature, driving the pathophysiology of HFpEF [19,43].

Beyond the studies supporting the mechanistic link between chronic hyperleptinemia and HFpEF, several epidemiological studies in humans have associated chronic leptin elevation with a higher prevalence of HFpEF, even after adjusting for multiple confounders. The study by Wannamethee et al. investigated the relationship between BMI, waist circumference, and the risk of HF in older men with and without pre-existing CAD, emphasizing the role of plasma leptin [44]. Over a nine-year follow-up of 4080 men aged 60 to 79 years, higher BMI was linked to a significantly increased HF risk in both groups, even after adjusting for cardiovascular risk factors. Leptin, an adipokine associated with obesity, was found to be a significant predictor of HF in men without pre-existing CHD, independent from BMI and other mediators, with an adjusted HR of 1.30 per 1-SD increase (*p* = 0.01). However, in men with pre-existing CAD, leptin showed no significant association with HF. Importantly, adjusting for leptin diminished the BMI-HF association in those without CAD; whereas, BMI remained a significant HF risk factor in men with CAD. The findings suggest that leptin may mediate the link between obesity and HF in the absence of CAD, but that obesity’s impact on HF in those with CAD is likely independent from leptin. This study underscores the need for further research to understand the different pathways through which obesity influences HF risk [44].

The study by Lieb et al. examined the relationship between plasma leptin levels and the risk of congestive HF, CVD, and all-cause mortality in elderly individuals, utilizing data from the Framingham Heart Study [45]. The study included 818 participants (mean age 79 years, 62% women), with a mean follow-up period of 8 years. Results showed that leptin levels were significantly higher in women and correlated strongly with BMI. In multivariable-adjusted models that excluded BMI, higher leptin levels were associated with an increased risk of HF and CVD. However, these associations were attenuated and became nonsignificant for HF and borderline for CVD when BMI was included in the models, suggesting that leptin does not provide incremental prognostic information beyond BMI. Interestingly, a U-shaped association was found between leptin levels and total mortality, indicating greater mortality risk at both low and high leptin levels. This association was primarily driven by non-cardiovascular causes. The study concludes that, although leptin is linked to HF and CVD, BMI remains a stronger predictor, and the complex relationship of leptin with mortality warrants further research to understand the underlying mechanisms [45].

Further insights into the role of leptin in HFpEF were revealed through a study involving 100 Egyptian patients with CAD and HFpEF, compared to 100 age- and sex-matched healthy controls [46]. The study found significantly higher serum leptin levels in HFpEF patients (26.1 ± 6.2 ng/mL) compared to controls (15.7 ± 3.3 ng/mL, *p* < 0.001). It identified the LEP AA genotype and LEPR RR genotype as critical risk factors, with HFpEF patients showing frequencies of 14% and 17%, respectively, compared to 4% (*p* = 0.02) and 6% (*p* = 0.007) in controls. The LEPR RR genotype was associated with a 3.7-fold increased risk (OR = 3.7, 95% CI: 1.4–10.3), while the LEP AA genotype carried a 3.9-fold risk (OR = 3.9, 95% CI: 1.2–12.6). Dyslipidemia was linked to elevated leptin levels, with HFpEF patients showing significantly higher total cholesterol (240.5 ± 27.4 mg/dL) and triglycerides (175.7 ± 13.9 mg/dL) and lower high-density lipoprotein cholesterol (41.8 ± 4.2 mg/dL, all *p* < 0.001). These findings suggest that leptin contributes to HFpEF through metabolic and inflammatory pathways, promoting arterial stiffness and endothelial dysfunction, while its neurohumoral activation and oxidative stress further exacerbate cardiac remodeling and functional decline [46].

The fact that leptin resistance and hyperaldosteronism are associated with HFpEF has been supported by a study in which researchers developed a murine model combining leptin-receptor-deficient (db/db) mice with chronic aldosterone infusion [47]. This “two-hit” model effectively recapitulates hallmark features of human HFpEF, including diastolic dysfunction, preserved LVEF, concentric LV hypertrophy, elevated plasma B-type natriuretic peptide (BNP), and extracardiac comorbidities, such as severe obesity, hyperglycemia, pulmonary edema, and vascular dysfunction. Neither db/db mice nor aldosterone infusion alone produced severe HFpEF phenotypes, but their combination synergistically induced diastolic impairments, characterized by prolonged Ca^2+^ transient decay, elevated diastolic intracellular Ca^2+^ levels, and delayed sarcoplasmic reticulum Ca^2+^ reuptake. At the cellular level, this model demonstrated arrhythmogenic remodeling, including action potential duration prolongation, the increased short-term variability of APD, enhanced late Na⁺ current (I_Na,late_), and delayed afterdepolarizations. Empagliflozin, a SGLT2i, reversed I_Na,late_ enhancement and arrhythmogenic action potential duration changes, suggesting a cardioprotective effect independent from SGLT2 expression in cardiomyocytes. This model underscores the synergistic role of metabolic dysregulation and mineralocorticoid excess in HFpEF pathophysiology and highlights therapeutic potential targeting these pathways, particularly in patients with diabetic HFpEF. These findings advance the understanding of HFpEF mechanisms and provide a platform for testing novel interventions [47].

The AtheroGene study demonstrated that elevated leptin concentrations are significantly associated with cardiovascular death and nonfatal myocardial infarction in women with CAD (HR: 1.32, 95% CI: 1.05–1.65, *p* = 0.02), while no significant association was found in men (*p* > 0.05). Key predictors of leptin concentration included BMI, renal function, and age, with women showing nearly four times higher median leptin levels than men (24.6 ng/mL vs. 6.6 ng/mL, *p* < 0.0005) [48].

Chronic hyperleptinemia in HFpEF patients is largely attributable to higher BMI, as obesity is both a driver of HFpEF pathogenesis and a key factor in increased leptin production. Furthermore, increased leptin in cardiometabolic HFpEF might also result from secretion by the epicardial adipose tissue [49]. In any case, as already analyzed, the resulting chronic hyperleptinemia leads to insulin resistance, chronic inflammation, diastolic dysfunction, myocardial remodeling, and arterial stiffness—processes central to HFpEF. According to a recent study, leptin levels in HFpEF are not significantly correlated with NT-proBNP, suggesting that leptin’s role may be more causative, reflecting systemic metabolic dysfunction rather than cardiac wall stress [50].

In contrast, HFrEF exhibits a reverse metabolic profile characterized by elevated leptin levels, even in a catabolic state, which interestingly correlates with better outcomes [21,50]. Chronic hyperleptinemia in HFrEF might be a result of chronic inflammation, resulting insulin resistance, ectopic leptin production from cardiomyocytes or vascular cells, and hypoxia-induced leptin production via various tissues due to hypoperfusion [40,51,52]. Interestingly, higher leptin levels in HFrEF are inversely correlated with NT-proBNP and associated with a lower risk of adverse outcomes; although, this significance disappears after adjusting for NT-proBNP. This relationship may reflect the “obesity paradox”, where higher leptin levels in non-cachectic patients are indicative of better nutritional status and prognosis [21,50].

The above aligns with the concept that HFpEF is potentially a metabolic-driven disease with chronic hyperleptinemia having a potential causative role early in the pathophysiology, which leads to myocardial dysfunction. On the other hand, the cause of HFrEF is usually an acute or chronic disease that leads to severe myocardial dysfunction as the first step, leading secondary to chronic hyperleptinemia, which further worsens cardiac function, leading to a vicious cycle.

Contrary to the above studies supporting the association between chronic hyperleptinemia and diastolic dysfunction, there is contradictory evidence, as highlighted by a recent cross-sectional study from the Genetic Epidemiology Network of Arteriopathy (GENOA) [53,54]. This study, conducted with 1172 black participants with preserved LVEF, found that higher plasma leptin levels were independently associated with lower LV mass and reduced myocardial stiffness (measured by diastolic wall strain, DWS) in obese women, after adjusting for confounders, such as BMI, blood pressure, and diabetes. These associations were not statistically significant in men. The protective associations observed in obese women might be explained by leptin’s ability to improve myocardial metabolism, reduce myocardial steatosis, and possibly, exert direct cardioprotective effects by modulating lipid accumulation in the heart, as already analyzed earlier in the manuscript [16]. It is possible that these effects differ based on population characteristics, such as sex, ethnicity, and obesity status, as well as its systemic versus tissue-specific actions. While these findings suggest a cardioprotective role of leptin in certain subgroups, further research is needed to confirm their generalizability and elucidate the underlying mechanisms [53,54].

Furthermore, the study by Martin et al. investigated whether higher baseline serum leptin levels are associated with an increased risk of CVD in a diverse cohort from the Multi-Ethnic Study of Atherosclerosis (MESA) [55]. The study included 1905 participants who were free of CVD at baseline, with a median follow-up period of 7.6 years. Leptin levels were analyzed as a log-transformed continuous variable, and multivariable Cox regression was used to assess associations with hard CVD events, including coronary heart disease and stroke. Results showed no statistically significant association between leptin levels and incident hard CVD in either women (HR per 1 SD increase in ln(leptin): 1.16, 95% CI 0.78–1.73, *p* = 0.46) or men (HR: 0.91, 95% CI 0.69–1.20, *p* = 0.51). When pooling sexes and adjusting for sex, age, and ethnicity, the association remained nonsignificant (HR: 0.98, 95% CI 0.78–1.23, *p* = 0.89). Adjusting for additional CVD risk factors did not change the outcome. Exploratory analyses showed a potential but nonsignificant signal for higher HF risk in women with elevated leptin, which disappeared after adjusting for traditional risk factors. The study concluded that, in this multi-ethnic cohort, baseline leptin levels were not significantly associated with future CVD risk. The findings suggest that leptin may not independently predict CVD or HF risk, highlighting the complexity of leptin’s role in cardiovascular health and the need for further research [55].

Targeting leptin in obesity, T2DM, and cardiometabolic disorders offers significant therapeutic potential by addressing leptin resistance and restoring its physiological functions. Two primary approaches can be considered.

▪ The first focuses on improving leptin sensitivity in the hypothalamus to promote satiety and in peripheral extracardiac tissues to enhance insulin sensitivity. Weight loss through restrictive diets and lifestyle modifications has been shown to improve leptin sensitivity, aiding in sustained weight management and reducing the risk of obesity-related comorbidities, including CVD. Leptin-sensitizing agents enhance leptin signaling and can counteract leptin resistance. Potential drugs include celastrol, which improves hypothalamic leptin signaling and energy expenditure via interleukin-1 receptor 1 (IL1R1) upregulation, and withaferin A, which reduces fat mass and improves leptin sensitivity by promoting LEP-R signaling and the signal transducer and activator of transcription 3 (STAT3) phosphorylation. Other agents, such as metformin, resveratrol, and glucagon-like peptide-1 receptor agonists (GLP-1RAs), restore endogenous leptin function and enhance anorectic (appetite-suppressing) effects. Additionally, inhibitors of leptin signaling suppressors, like the suppressor of cytokine signaling 3 (SOCS3) and protein tyrosine phosphatase 1B (PTP1B) (e.g., trodusquemine), show promise in preclinical models. Strategies to improve leptin transport across the blood–brain barrier or modulate leptin receptor endocytosis also offer potential. While some therapies, such as metreleptin combined with pramlintide, have shown weight loss benefits, challenges include variability in clinical efficacy, adverse effects, and the need for further research to optimize therapeutic use in hyperleptinemic patients [18,56].
▪ The second approach focuses on inhibiting leptin signaling specifically in cardiovascular tissues. The development of cardioselective leptin receptor antagonists holds potential for mitigating the adverse effects of chronic hyperleptinemia on the cardiovascular system. However, since leptin also exerts beneficial effects on cardiac metabolism and lipid regulation, interventions targeting leptin’s actions in the heart must be approached with caution. This is particularly important considering the findings from the GENOA study, which underscore the complex and context-dependent roles of leptin in cardiovascular health [18,54,55].

The key cardiovascular effects of chronic hyperleptinemia implicated in HFpEF pathophysiology are presented in Table 1.

### 4.2. Adiponectin

Adiponectin is a 30 kDa adipokine secreted primarily by adipose tissue that exerts its effects through the activation of two main receptors, AdipoR1 and AdipoR2, on various cell types. These receptors trigger distinct intracellular signaling cascades critical for adiponectin’s physiological actions. AdipoR1, predominantly expressed in skeletal muscle, exhibits a high affinity for the globular form of adiponectin and activates AMP-activated protein kinase (AMPK). This pathway enhances fatty acid oxidation, glucose uptake, and energy homeostasis. AdipoR2, primarily located in the liver, activates both AMPK and PPAR-α signaling pathways, reducing gluconeogenesis and promoting fatty acid oxidation. Additionally, adiponectin interacts with adaptor protein APPL1, facilitating the downstream activation of these pathways and further regulating insulin sensitivity and lipid metabolism. Through these mechanisms, adiponectin modulates inflammation, improves metabolic efficiency, and protects against metabolic diseases, including obesity and T2DM [58,59].

Adiponectin is widely recognized as an anti-inflammatory adipokine with beneficial effects on metabolic and cardiovascular health. It exerts its anti-inflammatory properties by downregulating pro-inflammatory markers and reducing oxidative stress, thereby improving insulin sensitivity and offering protection against atherosclerosis. Evidence suggests a strong inverse relationship between circulating adiponectin levels and inflammatory markers, such as CRP, IL-6, and TNF-α. This adipokine directly modulates endothelial inflammation by suppressing the expression of adhesion molecules and inhibiting the production of pro-inflammatory cytokines, primarily through the negative regulation of NF-κB signaling pathways. These mechanisms underscore its critical role in mitigating vascular inflammation and preserving endothelial function, which are significantly implicated in the pathophysiology of cardiometabolic HFpEF [41,60].

In T2DM mouse studies, adiponectin was shown to suppress TNF-α expression, while TNF-α inhibition correspondingly elevated adiponectin levels in coronary arterioles and the aorta. This bidirectional interaction highlights adiponectin’s capacity to counteract TNF-α-mediated endothelial dysfunction and potentially avert vascular injury in T2DM. Furthermore, studies in mice and humans have demonstrated that hypoadiponectinemia—a condition of abnormally low adiponectin levels—is associated with impaired endothelial function in both animal models and humans. Conversely, adiponectin supplementation in obese animal models has been shown to enhance ΝO production and endothelial ΝO synthase (eNOS) phosphorylation, effectively reversing endothelial dysfunction [61,62,63,64,65].

A particularly intriguing aspect of adiponectin’s role in vascular health involves its association with the NOD-, LRR-, and pyrin-domain-containing protein 3 (NLRP3) inflammasome, a critical component of the innate immune response. Hypoadiponectinemia has been linked to the activation of the NLRP3 inflammasome in diabetic vascular endothelial dysfunction. This activation triggers caspase-1, which promotes the release of pro-inflammatory cytokines, such as IL-1β and IL-18, contributing to chronic inflammation and the progression of T2DM, atherosclerosis, and cardiometabolic HFpEF [66,67].

Further evidence implicating adiponectin in HFpEF pathophysiology emerges from a preclinical study highlighting its role in coronary angiogenesis and endothelial function. The findings reveal that oxidative stress, driven by elevated 4-hydroxy-2-nonenal (4HNE), induces adiponectin resistance in coronary endothelial cells (CECs), thereby impairing coronary angiogenesis—a critical factor contributing to microvascular dysfunction in diabetic HFpEF. The worsening of these effects with ALDH2 inhibition and their reversal through ALDH2 activation (Alda1) underscores the importance of adiponectin signaling in preserving coronary microvascular integrity. Furthermore, the pronounced diastolic dysfunction and increased adiponectin resistance observed in diabetic mice with low ALDH2 activity (AF mice) reinforce the link between adiponectin dysfunction and HFpEF progression. By restoring adiponectin-mediated angiogenesis, ALDH2 activation mitigates oxidative-stress-induced impairments, providing a promising therapeutic approach that targets the microvascular underpinnings of HFpEF and offering valuable insights into its pathophysiology and potential treatment strategies [68].

In the myocardium, adiponectin prevents pathological remodeling by inhibiting hypertrophy and fibrosis. Cardiomyocyte hypertrophy, a process driven by ET-1 and α-adrenergic stimulation, is attenuated by adiponectin through AMPK-mediated inhibition of extracellular signal-regulated kinase (ERK) activation. Furthermore, adiponectin’s anti-fibrotic effects are partly attributed to the inhibition of angiotensin-II-induced cardiac fibrosis via PPAR-α activation. This pathway reduces the deposition of extracellular matrix proteins, thereby mitigating myocardial stiffening—a key contributor to HFpEF pathophysiology. Additionally, adiponectin has been shown to decrease ROS production and improve calcium handling in cardiomyocytes, supporting better diastolic function and overall myocardial efficiency [66,69,70,71].

In patients with CAD or HFpEF, reduced adiponectin levels are accompanied by elevated pro-inflammatory mediators, including IL-6, TNF-α, and TLR-4. These inflammatory alterations contribute to the pathological processes including atherosclerosis, myocardial fibrosis, myocardial hypertrophy, and diastolic dysfunction [41].

Preclinical studies provide compelling evidence for adiponectin’s protective cardiovascular role. A study investigating the impact of adiponectin deficiency in aldosterone-induced hypertension and HFpEF demonstrated its critical involvement in mitigating cardiac remodeling and diastolic dysfunction. In this study, wild-type (WT) and adiponectin-deficient (APNKO) mice were subjected to aldosterone infusion, uninephrectomy, and a high-salt diet over four weeks [72]. Results showed that aldosterone infusion significantly increased blood pressure in WT mice from 109 ± 3 mm Hg to 132 ± 2 mm Hg (*p* < 0.01), with a further rise in APNKO mice to 140 ± 3 mm Hg (*p* < 0.05 vs. aldosterone-treated WT), highlighting the role of adiponectin in modulating hypertensive responses. LV hypertrophy, as assessed by the LV-to-body weight ratio, was significantly increased in aldosterone-treated WT mice (4.8 ± 0.2 mg/g) compared to untreated WT controls (4.1 ± 0.2 mg/g), and further exacerbated in aldosterone-treated APNKO mice (6.0 ± 0.4 mg/g, *p* < 0.05 for both comparisons), indicating aggravated cardiac remodeling in the absence of adiponectin. Although LVEF was preserved in both groups, diastolic dysfunction metrics, including the E/A and E/e′ ratios, were significantly worsened in APNKO mice compared to aldosterone-treated WT mice (*p* < 0.05 for both), accompanied by greater pulmonary congestion (*p* < 0.01). On a molecular level, aldosterone increased matrix metalloproteinase-2 expression in WT hearts (*p* < 0.05 vs. WT controls, *p* < 0.01 vs. APNKO), while pro-inflammatory markers, such as atrial natriuretic peptide (ANP), interferon-γ, and TNF-α, were significantly upregulated in aldosterone-treated WT hearts and further elevated in aldosterone-treated APNKO mice (*p* < 0.01 for all comparisons). These findings underscore adiponectin’s role in protecting against hypertension-induced diastolic dysfunction and LV hypertrophy, primarily by attenuating inflammation and stress responses [72].

Further evidence for the implications of adiponectin in HFpEF comes from another mechanistic preclinical study, which demonstrated that chronic adiponectin overexpression and supplementation significantly mitigated the progression of aldosterone-induced HFpEF, independent from blood pressure [73]. Key findings include a reduction in LV hypertrophy, as reflected by a lower total wall thickness (0.93 ± 0.02 mm vs. 1.04 ± 0.02 mm, *p* < 0.01) and decreased LV mass (110.9 ± 12.7 mg vs. 140.4 ± 6.5 mg, *p* < 0.05) in adiponectin transgenic (APNTG) mice compared to wild-type (WT) aldosterone-infused mice. Diastolic function was notably improved, with a lower E/A ratio (1.50 ± 0.09 vs. 2.17 ± 0.17, *p* < 0.01), normalized deceleration time (20.42 ± 0.77 ms vs. 15.56 ± 1.00 ms, *p* < 0.01), and reduced E/e′ ratio (42.67 ± 1.28 vs. 56.84 ± 1.96, *p* < 0.01). Furthermore, myocardial oxidative stress was attenuated, as evidenced by a 54% decrease in nitrotyrosine staining (*p* < 0.05). Mechanistically, adiponectin preserved phosphorylation of PLB at Ser16, thereby improving calcium handling without altering SERCA2a protein expression. These results suggest that targeting the adiponectin signaling pathway may offer a novel therapeutic approach to manage HFpEF by addressing myocardial remodeling, oxidative stress, and diastolic dysfunction [73].

In addition to preclinical studies, low adiponectin has also been associated with early diastolic dysfunction in humans. A case-control study of 25 patients with diastolic dysfunction and 25 age-matched controls demonstrated significantly lower plasma levels of total adiponectin (median 4.4 μg/mL vs. 12.7 μg/mL, *p* = 0.001), high molecular weight (HMW) adiponectin (median 1.3 μg/mL vs. 3.4 μg/mL, *p* = 0.02), and mid-to-low molecular weight (MMW + LMW) adiponectin (median 3.8 μg/mL vs. 7.2 μg/mL, *p* = 0.01) in the DD group. Linear regression analysis revealed an independent association of diastolic dysfunction with BMI (*p* < 0.05), total adiponectin (*p* < 0.001), HMW adiponectin (*p* = 0.03), and MMW + LMW adiponectin (*p* = 0.004), even after adjusting for age, sex, and blood pressure. Additionally, BMI correlated negatively with total adiponectin (r = −0.46, *p* = 0.003), HMW adiponectin (r = −0.32, *p* = 0.038), and MMW + LMW adiponectin (r = −0.40, *p* = 0.006). These findings support the hypothesis that adiponectin deficiency, possibly through oxidative stress and reduced ΝO bioavailability, contributes to impaired myocardial relaxation and the pathogenesis of diastolic dysfunction, linking adiposity with cardiovascular dysfunction [74].

An additional clinical study, low adiponectin is associated with diastolic dysfunction in women: a cross-sectional study from the Tromsø Study, explored the relationship between adiponectin levels and diastolic dysfunction in a large cohort of 1165 women and 896 men without diabetes [75]. The results revealed significant sex differences in adiponectin’s association with diastolic dysfunction indices. In women, decreased adiponectin levels were linked to higher odds of average e’ wave < 9 cm/s (OR 1.17 per 1 μg/mL decrease, 95% CI 1.04–1.30) and an E/e’ ratio ≥ 8 (OR 1.12 per 1 μg/mL decrease, 95% CI 1.02–1.24). Furthermore, low adiponectin was associated with increased odds of concentric LV hypertrophy (OR 2.44, 95% CI 1.03–5.77) and moderately enlarged left atria (OR 1.43, 95% CI 1.01–2.03) in women. Interestingly, in men, low adiponectin levels correlated with lower odds of concentric (OR 0.32, 95% CI 0.11–0.88) and eccentric hypertrophy (OR 0.53, 95% CI 0.33–0.88). These findings highlight a nonlinear relationship between adiponectin and LV mass in women, suggesting a complex, sex-specific interaction between adiponectin and cardiac remodeling [75].

Although elevated adiponectin levels are typically regarded as beneficial for metabolic health, there is a U-shaped correlation between adiponectin levels and health outcomes, where both low and excessively high levels have been associated with various disease states [14,75,76]. This phenomenon, known as the “adiponectin paradox”, highlights the complex role of adiponectin in cardiovascular health. While adiponectin is generally considered protective, its elevated levels in patients with worsening HF present a contradiction. In HF, increased adiponectin levels may reflect either a compensatory response to cardiac dysfunction or a maladaptive state characterized by adiponectin resistance. As a result, while adiponectin’s anti-inflammatory and cardioprotective properties are well-documented, elevated adiponectin levels in HF patients are often a marker of disease severity rather than an indicator of resilience [14,76,77].

The adiponectin paradox in HF patients is greatly highlighted by a recent meta-analysis by Bai et al., which evaluated the prognostic significance of elevated circulating adiponectin levels in HF [76]. This systematic review and meta-analysis included seven studies involving 862 HF patients and demonstrated that higher adiponectin levels were associated with significantly worse outcomes. Specifically, patients with elevated adiponectin levels had a 2.05-fold increased risk of all-cause mortality (relative risk [RR] 2.05; 95% CI 1.22–3.43; *p* < 0.05) and a 2.22-fold increased risk of combined endpoints of death and readmission (RR 2.22; 95% CI 1.38–3.57; *p* < 0.05). Importantly, the analysis revealed substantial heterogeneity for all-cause mortality outcomes (I^2^ = 70.5%; *p* = 0.005) but no significant heterogeneity for the combined endpoints (I^2^ = 0%; *p* = 0.522). These findings underscore that elevated adiponectin, rather than being purely protective, may signify advanced disease severity and metabolic derangements, offering a potential biomarker for improved risk stratification in HF management [76].

Adiponectin resistance offers a potential explanation for the adiponectin paradox, as demonstrated by Khan et al. in their study on advanced HF patients undergoing ventricular assist device (VAD) implantation [77]. The study found significantly elevated serum adiponectin levels in HF patients pre-VAD compared to healthy controls (13.3 ± 4.9 μg/mL vs. 6.4 ± 2.1 μg/mL, *p* = 0.02). These levels normalized post-VAD (7.4 ± 3.4 μg/mL, *p* < 0.05), correlating with improved insulin resistance (HOMA-IR reduced from 6.3 ± 5.8 to 3.6 ± 2.9, *p* < 0.05). Moreover, adiponectin receptor expression, suppressed in failing myocardium, increased significantly after VAD support, indicating a reversal of adiponectin resistance. The study also revealed that adipose tissue inflammation, characterized by a 25% increase in macrophage infiltration (*p* < 0.01) and reduced adipocyte size in HF patients, normalized after mechanical unloading. These findings suggest that elevated adiponectin levels in HF may reflect a compensatory yet insufficient response to systemic inflammation and oxidative stress. Mechanical unloading not only reversed inflammation but also improved adiponectin signaling, offering insights into the paradoxical role of adiponectin in advanced HF and potential therapeutic strategies to restore its beneficial effects [77].

The interplay between adiponectin and natriuretic peptides adds another layer of complexity. Natriuretic peptides enhance adiponectin production, and their elevation in HF could be a mechanism to counteract cardiac and vascular stress. BNP and adiponectin share beneficial effects, including vasodilation and reduced sympathetic activity, yet the simultaneous rise in both biomarkers may indicate advanced cardiac dysfunction rather than effective compensation. Studies have shown that adiponectin’s elevation is often accompanied by higher BNP levels, reflecting severe hemodynamic compromise and a higher risk of adverse events [78,79].

The adiponectin paradox extends beyond patients with HF, as highlighted by findings from the Dallas Heart Study, a large, multiethnic cohort of relatively young adults (mean age 43.4 years) without baseline CVD [80]. Over a median follow-up of 10.4 years, higher adiponectin quartiles were independently associated with increased risks of adverse outcomes. Compared to the lowest quartile (Q1), the highest adiponectin quartile (Q4) was associated with significantly elevated hazards for all-cause mortality (HR 2.27; 95% CI 1.47–3.50; *p* = 0.0002), CVD mortality (HR 2.43; 95% CI 1.15–5.15; *p* = 0.02), major adverse cardiovascular and cerebrovascular events (MACCE) (HR 1.71; 95% CI 1.13–2.60; *p* = 0.01), and HF (HR 2.95; 95% CI 1.14–7.67; *p* = 0.03), after adjusting for potential confounders, such as age, sex, race, BMI, hypertension, diabetes, and inflammatory markers. These associations persisted across subgroups stratified by age, sex, race, metabolic syndrome, and other cardiometabolic factors. Interestingly, while higher adiponectin levels were linked to a favorable cardiometabolic profile (e.g., lower BMI, improved high-density lipoprotein cholesterol, and reduced liver fat), they paradoxically correlated with worse outcomes, suggesting a potential role for adiponectin resistance even in metabolically healthy individuals. This resistance may diminish the regulatory actions of adiponectin, or elevated levels may reflect compensatory responses to systemic inflammation or neurohormonal activation, as indicated by the attenuation of associations when adjusted for NT-proBNP levels. These findings underscore the complexity of adiponectin’s role in health and disease and suggest that variations in adiponectin resistance, even in individuals without overt CVD, might drive these adverse outcomes. Further research is needed to elucidate the underlying mechanisms and assess whether targeting adiponectin pathways could modify risk [79].

Adiponectin, like leptin, provides further evidence that HFpEF is primarily a metabolically driven condition, distinct from HFrEF. As already stated, studies have shown that adiponectin levels are significantly elevated in HFrEF, correlating positively with NT-proBNP and adverse outcomes. A recent study showed that the association with adverse outcomes loses statistical significance after adjusting for NT-proBNP, further supporting that adiponectin is more of a marker of disease severity rather than a direct contributor to outcomes. In contrast, according to the same study, HFpEF patients exhibited adiponectin levels comparable to non-HF controls [50]. This phenomenon may result from the obesity-associated suppression of adiponectin, which prevents the elevations typically seen in HFrEF. This is similar to the well-known phenomenon that natriuretic peptides are lower in obese patients.

Therapeutic strategies targeting adiponectin hold promise for improving metabolic profiles and cardiovascular health, particularly in conditions characterized by decreased adiponectin levels, such as atherosclerosis, insulin resistance, and CVD [65]. Lifestyle modifications, including weight management, low-carbohydrate diets, and moderate physical activity, are well-established interventions that increase adiponectin levels. Pharmacological approaches have also shown potential; for instance, the PPAR-α agonist fenofibrate enhances adiponectin levels and insulin sensitivity in patients with hypertriglyceridemia or metabolic syndrome. However, lipid-lowering agents, like statins (e.g., simvastatin and rosuvastatin), paradoxically reduce adiponectin levels, while improving endothelial function. In contrast, antihypertensive agents, including amlodipine, candesartan, and ramipril, significantly boost adiponectin levels compared to placebo or thiazides. Emerging therapies, such as GLP-1RA and SGLT2i, also demonstrate favorable effects by reducing inflammation, improving endothelial function, and enhancing insulin sensitivity, potentially interacting with adiponectin signaling [65].

AdipoR agonists, particularly AdipoRON, an orally active agent, have demonstrated potent anti-diabetic, anti-inflammatory, renoprotective, and cardiovascular benefits in preclinical models, with promising results in addressing HFpEF [81]. In a study utilizing a two-hit HFpEF mouse model induced by a high-fat diet (60%) combined with L-NAME drinking water, AdipoRON (50 mg/kg) was administered daily by gavage for four weeks. Echocardiographic analysis revealed that AdipoRON improved cardiac function and alleviated HFpEF phenotypes through mechanisms involving reduced lipid droplet accumulation and fibrosis. Specifically, AdipoRON upregulated AdipoR1/2 independently from adiponectin and restored lipid homeostasis by promoting fatty acid oxidation and balancing myocardial fatty acid intake and transport. Key molecular findings indicated that AdipoRON activated downstream AMPK-α and PPAR-α signaling pathways, which were critical to its cardioprotective effects. The beneficial effects were reversed upon the inhibition of AMPK-α with compound C (*p* < 0.01) and PPAR-α with GW6471 (*p* < 0.01), confirming the dependence on these pathways. Untargeted metabolomics demonstrated significant restoration of lipid metabolism, while RNA sequencing and molecular analyses further supported the therapeutic role of AdipoRON in reducing fibrosis and lipid accumulation. These findings highlight AdipoRON’s capacity to mitigate mechanical and metabolic stressors in HFpEF by improving fatty acid oxidation and reducing pathological fibrosis, underscoring its therapeutic potential. Further validation in human trials is warranted to explore its broader applicability in metabolic and CVD [81].

The key cardiovascular effects of adiponectin implicated in HFpEF pathophysiology are presented in Table 2.

### 4.3. Resistin

Resistin is a 12.5 kDa, cysteine-rich adipokine that was initially identified as a critical factor linking metabolic dysfunction to CVD and insulin resistance. In rodents, it is primarily secreted by white adipocytes. In humans, however, it is mainly expressed by lymphocytes, monocytes, and macrophages but is also produced in smaller amounts in other tissues, while its expression in the adipose tissue is due to non-adipocyte cells. Despite these differences, resistin’s role in metabolic and CVD has been widely studied and is recognized as a key mediator of inflammation and metabolic disturbances associated with obesity and T2DM. Elevated resistin levels have been shown to promote endothelial dysfunction, VSMC proliferation, and oxidative stress, which contribute to the development of hypertension, atherosclerosis, and other CVDs. These effects, along with its association with insulin resistance, increased hepatic gluconeogenesis, and dyslipidemia, position resistin as a potential therapeutic target for metabolic and cardiovascular conditions, including HFpEF [5,82].

Resistin exerts its biological effects by interacting with specific receptors and activating intracellular signaling pathways [82,83]. One key receptor identified for human resistin is adenylate cyclase-associated protein-1 (CAP-1). The central proline-rich Src homology (SH3) domain of CAP-1 binds resistin, initiating a cascade of signaling events. Upon binding, resistin upregulates the expression of NF-κB, a pivotal transcription factor that mediates inflammatory responses. This activation is accompanied by an increase in cyclic adenosine monophosphate levels and the activation of protein kinase A, which further amplifies the inflammatory response. Resistin binding also stabilizes the mRNA of various pro-inflammatory cytokines, enhancing their secretion. In addition to CAP-1, resistin has been proposed to interact with TLR-4, competing for binding with lipopolysaccharides. This interaction contributes to the activation of downstream inflammatory pathways, similar to those triggered by TLR-4 signaling during immune responses. Furthermore, resistin has been implicated in modulating the NF-κB pathway through crosstalk with other signaling molecules, including MAPKs and ERK1/2, which are essential for cellular responses to stress and inflammation. In macrophages, monocytes, and hepatic stellate cells, resistin stimulates the secretion of pro-inflammatory cytokines, such as TNF-α, IL-1β, IL-6, IL-8, and IL-12. Resistin also induces the secretion of monocyte chemotactic protein-1, further promoting inflammatory cell recruitment and amplifying systemic inflammation [5,14,41,82,83].

In endothelial cells, resistin diminishes NO bioavailability by increasing ROS production and elevating ET-1 levels, a potent vasoconstrictor. This impairs vasodilation and promotes endothelial dysfunction, a precursor to atherosclerosis and vascular complications observed in HFpEF patients. Additionally, resistin upregulates adhesion molecules, such as vascular cell adhesion molecule-1 and intercellular adhesion molecule-1, facilitating leukocyte infiltration and further perpetuating vascular inflammation. Collectively, these mechanisms outline resistin’s role in promoting a pro-inflammatory, pro-thrombotic, and insulin-resistant environment, which significantly contributes to cardiovascular complications, including cardiometabolic HFpEF [5,14,41,82,83].

In a study investigating resistin levels and their association with inflammatory and endothelial dysfunction markers in obese, postmenopausal women with T2DM, resistin levels were significantly higher in women with coronary heart disease (CHD) compared to those without CHD (12.43 ± 5.21 ng/mL vs. 9.50 ± 3.33 ng/mL, *p* < 0.001). This difference remained significant after age adjustment (*p* = 0.013). Resistin levels were independently associated with markers of systemic inflammation and endothelial dysfunction, including IL-6, high-sensitive CRP, and soluble vascular cell adhesion molecule (sVCAM), with sVCAM showing the strongest association (*p* = 0.009). Multivariate analysis revealed that creatinine, triglycerides, high-sensitive CRP, and IL-6 were significant determinants of resistin concentrations [83].

Resistin’s cardiovascular impact is profound and multifactorial, with direct effects on cardiac structure and function. Chronic hyper-resistinemia has been implicated in the pathogenesis of myocardial hypertrophy and fibrosis, both of which are hallmarks of HFpEF. The hormone acts on cardiac fibroblasts to stimulate collagen synthesis and deposition, leading to myocardial stiffness and impaired diastolic relaxation. Resistin-induced fibrosis is mediated through the activation of pro-fibrotic signaling cascades, including the TGF-β/Smad pathway, and is accompanied by the increased expression of matrix metalloproteinases that alter extracellular matrix remodeling [84,85,86,87].

A recent preclinical study has explored myocardial fibrosis in HFpEF using a mouse model induced by transverse aortic constriction and deoxycorticosterone acetate pellet implantation, identifying key fibrosis-related genes and pathways [88]. Myocardial fibrosis, a critical contributor to diastolic dysfunction and ventricular stiffness in HFpEF, was significantly increased in the HFpEF group, as demonstrated by histological staining (*p* < 0.05 for fibrosis area). Bioinformatics analysis revealed nine significantly enriched fibrosis-related pathways, with 112 differentially expressed genes identified, including resistin-like molecule gamma (RELM-γ) and adenylate cyclase type 1 (Adcy1). In HFpEF mice, RELM-γ was significantly upregulated (logFC = 3.4, corrected *p* = 5.79 × 10^−6^), promoting fibrosis through increased collagen synthesis and extracellular matrix deposition, while Adcy1 was significantly downregulated (logFC = 0.95, corrected *p* = 4.32 × 10^−6^), with an inhibitory role in fibrosis regulation. These findings suggest that RELM-γ is a pro-fibrotic factor in HFpEF, highlighting its potential as a therapeutic target for reducing fibrosis and improving diastolic function in HFpEF [88].

Resistin also disrupts intracellular calcium homeostasis in cardiomyocytes, impairing myocardial contractility and lusitropy. By interfering with SERCA2a and enhancing calcium leak from the ryanodine receptor, resistin reduces the efficiency of diastolic calcium reuptake, contributing to diastolic dysfunction—a characteristic feature of HFpEF. Furthermore, resistin exacerbates oxidative stress within cardiac tissues by upregulating nicotinamide adenine dinucleotide phosphate oxidase activity, leading to mitochondrial dysfunction and increased ROS production. This oxidative stress accelerates cardiomyocyte apoptosis and tissue damage, worsening the cardiac remodeling process [87,89,90,91].

In addition to its fibrotic and contractile effects, resistin influences neurohormonal pathways. It enhances sympathetic nervous system activity, elevating blood pressure and heart rate, which increase myocardial afterload and further stress the heart. Resistin also modulates the renin–angiotensin–aldosterone system, promoting hypertensive responses and aggravating HF progression Animal models have provided strong evidence of resistin’s impact: studies involving adenoviral overexpression of resistin demonstrated significant myocardial hypertrophy, impaired LV contractility, and fibrosis in rats, mirroring the pathological features observed in HFpEF patients [91,92,93,94,95].

Preclinical models have been pivotal in elucidating resistin’s cardiac effects. For instance, adenoviral-mediated overexpression of resistin in rodents led to marked cardiac dysfunction characterized by hypertrophy, impaired contractility, and pronounced myocardial fibrosis [90,91,92]. These models revealed that resistin exacerbates oxidative stress and activates apoptotic signaling pathways, contributing to cardiomyocyte death and further impairing cardiac function. Additionally, transgenic mice engineered to overexpress human resistin displayed significant cardiac hypertrophy, fibrosis, and diastolic dysfunction, reinforcing the hormone’s role in adverse cardiac remodeling [94,95].

In studies examining myocardial infarction models, resistin was found to localize specifically to areas of cardiac injury, where it exacerbated fibrosis and inflammation. These observations support the hypothesis that resistin acts as a stress-responsive cytokine, amplifying cardiac damage under ischemic conditions. Interestingly, pressure overload models demonstrated that resistin expression was significantly higher in fibrotic cardiac tissues compared to non-fibrotic regions, further linking resistin to pathological cardiac remodeling [94,95,96].

Despite the above evidence from preclinical mechanistic studies, clinical evidence does not support a strong connection of resistin with HFpEF. A recently published study analyzed data from the Multi-Ethnic Study of Atherosclerosis (MESA) to investigate the relationship between resistin levels and incident HF subtypes over a median follow-up of 10.5 years [97]. Elevated resistin levels were significantly associated with incident HF (HR 1.44, CI 1.18–1.75, *p* = 0.001) and specifically with HFrEF (HR 1.47, CI 1.07–2.02, *p* = 0.016). However, no significant association was found between resistin levels and HFpEF (HR 1.25, CI 0.89–1.75, *p* = 0.195). Resistin was not significantly associated with biomarkers, such as NT-proBNP and high-sensitive cardiac troponin T, or with myocardial fibrosis measures derived from cardiac magnetic resonance, including extracellular volume fraction and myocardial scar. Interestingly, resistin levels were negatively associated with post-contrast T1 times at 12 min (*p* = 0.022) and 25 min (*p* = 0.026). These findings highlight that resistin’s role as a biomarker appears more relevant to HFrEF than HFpEF, with no evident link to subclinical myocardial fibrosis or traditional biomarkers of HF. Resistin may serve as an early indicator of HF risk, particularly in populations predisposed to systolic dysfunction, emphasizing the molecular and pathophysiological differences between HFrEF and HFpEF. Further research is needed to explore resistin’s potential as a therapeutic target and its implications in cardiac remodeling [97].

The key cardiovascular effects of resistin implicated in HFpEF pathophysiology are presented in Table 3.

**Figure 1:**
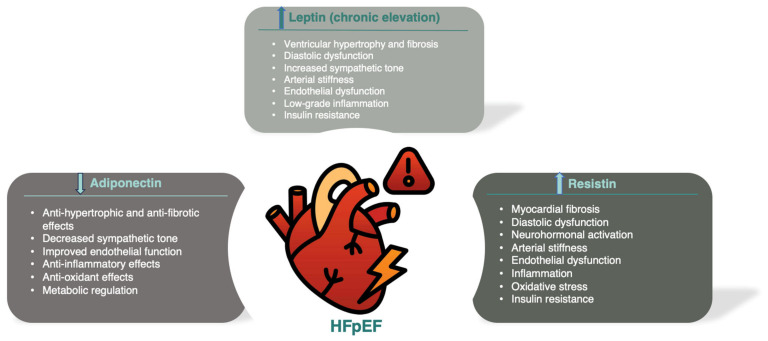
Key cardiovascular effects of the major adipokines implicated in HFpEF pathophysiology.

The key cardiovascular effects of the major adipokines (leptin, adiponectin, and resistin) implicated in HFpEF pathophysiology are illustrated in Figure 1.

### 4.4. Implications in Exercise Capacity in HFpEF

The study by Ramirez et al. meticulously examines the influence of various markers, including adipokines, on exercise capacity in 509 HFpEF patients, focusing on leptin, adiponectin, and resistin. They discovered that heightened leptin levels significantly correlated with diminished exercise tolerance, evidenced by a stark decrease in peak oxygen uptake (VO2). For every one standard deviation increment in leptin, there was a consequential 2.35 mL/kg/min reduction in peak VO2 (β = −2.35, *p* < 0.001), illustrating leptin’s negative impact on cardiovascular fitness, potentially through its pro-inflammatory actions and contribution to adverse ventricular remodeling [57].

Conversely, adiponectin demonstrated a protective effect, where an increase in its levels was positively associated with improved exercise performance. Specifically, each standard deviation increase in adiponectin was linked to a 0.74 mL/kg/min increase in peak VO2 (β = 0.74, *p* < 0.001), highlighting its potential role in enhancing cardiovascular health and mitigating HFpEF’s impact. This suggests that adiponectin might offset the negative influences of other adipokines, like leptin and resistin, in addition to reducing systemic inflammation [57].

Regarding resistin, the findings showed that higher levels were linked to poorer exercise capacity. Specifically, for each one standard deviation increase in resistin levels, there was a reduction of 0.86 mL/kg/min in peak VO2 (β = −0.86, *p* < 0.001). This suggests that resistin, similar to leptin, may have a negative impact on cardiovascular health, potentially through its involvement in inflammatory pathways that contribute to the pathophysiology of HFpEF. These data indicate that resistin, while not as impactful as leptin, still plays a significant role in the modulation of exercise capacity in patients with cardiovascular dysfunction [57].

The findings propose that therapeutic strategies targeting these adipokine pathways, particularly modulating leptin and boosting adiponectin, could potentially improve cardiac function and exercise tolerance in individuals suffering from obesity-related cardiovascular dysfunction. This paves the way for future interventions aimed at manipulating these adipokines to alleviate the adverse cardiac effects prevalent in HFpEF [97].

### 4.5. Other Adipokines

In addition to leptin, adiponectin, and resistin, there are other, less documented adipokines that significantly impact HFpEF.

A recently published study has demonstrated that elevated serum RBP-4, an adipokine with adverse cardiovascular effects, is associated with worse outcomes in elderly patients with chronic HF [98]. RBP-4 levels were significantly higher in HF patients compared to controls (46.66 ± 12.38 μg/mL vs. 40.71 ± 7.28 μg/mL, *p* < 0.001) and increased with worsening cardiac function, as indicated by higher New York Heart Association class, and reduced LVEF. Specifically, RBP-4 levels were negatively correlated with LVEF (r = −0.154, *p* < 0.001) and positively correlated with NT-proBNP (r = 0.074, *p* = 0.023). Subgroup analysis revealed RBP-4 concentrations of 43.70 ± 9.15 μg/mL in HFpEF, 45.32 ± 11.19 μg/mL in HF with mid-range ejection fraction (HFmrEF), and 48.73 ± 13.39 μg/mL in HFrEF (*p* < 0.001). Multivariate Cox regression identified log RBP4 as an independent predictor of major adverse cardiac events (HR = 2.61, 95% CI: 1.19–5.70, *p* = 0.016) and cardiovascular mortality (HR = 2.24, 95% CI: 1.35–5.39, *p* = 0.021). Kaplan–Meier survival analysis confirmed that high RBP-4 levels significantly increased the risk of adverse outcomes, highlighting its potential as a prognostic marker for risk stratification in HF, with notable implications for both HFpEF and HFrEF populations [98].

Another recently published study investigated the association between omentin-1 levels and HFpEF in Chinese elderly patients [99]. Plasma omentin-1 levels were significantly lower in HFpEF patients compared to controls (14.02 ± 8.35 vs. 19.74 ± 8.45 ng/mL, *p* < 0.001), while NT-proBNP, TNF-α, and IL-6 levels were significantly higher (*p* < 0.001). Omentin-1 levels were negatively correlated with NT-proBNP (r = −0.273, *p* < 0.001), TNF-α (r = −0.221, *p* = 0.001), and E/e’ (r = −0.340, *p* < 0.001), indicating its relationship with diastolic dysfunction and systemic inflammation. Multivariate logistic regression identified omentin-1 as an independent protective factor for HFpEF (odds ratio = 0.948, 95% CI 0.905–0.993, *p* = 0.025), while TNF-α, IL-6, and NT-proBNP were risk factors. ROC analysis demonstrated that omentin-1 had good diagnostic value for HFpEF (area under the curve [AUC] = 0.734, 95% CI 0.667–0.802), particularly in patients aged 70–80 years, where its predictive capability (AUC = 0.809) surpassed that of NT-proBNP (AUC = 0.674). These findings suggest that omentin-1 may serve as a novel biomarker for HFpEF diagnosis and progression, linked to its anti-inflammatory and cardioprotective properties [99].

Regarding additional adipokines, vaspin, an adipokine with anti-inflammatory properties, may halt cardiac degeneration and fibrosis, showing promise in experimental models of chronic HF. Visfatin, although lower in HF patients, is implicated in cardiovascular health due to its insulin-mimetic properties. Lipocalin-2 is linked to innate immune responses, suggesting its involvement in the pathogenesis of HF. Follistatin-like 1 is elevated in HFrEF and is associated with increased LV mass, potentially offering new therapeutic targets for HFpEF. Each of these adipokines offers a unique insight into the complex interplay between metabolic signals and cardiovascular pathology [100].

## 5. Gaps in Evidence and Future Research Directions

Another critical area is the development of specific inhibitors or modulators of adipokines. Research into drug development, perhaps focusing on small molecule inhibitors, peptide disruptors, or monoclonal antibodies that can specifically modulate the adipokine activity, could provide new treatment options for managing HFpEF.

Despite significant advancements in understanding the role of adipokines in the pathophysiology of HFpEF, substantial gaps remain that hinder the translation of these findings into clinical practice. One of the foremost challenges is the considerable variability in adipokine responses among individuals, which suggests a complex interplay of genetic, environmental, and lifestyle factors that are not yet fully elucidated. Future research should focus on personalized medicine approaches, leveraging genomics, proteomics, metabolomics, and other multi-omics technologies to predict individual responses to therapies targeting adipokine pathways. Such approaches could help identify specific patient subgroups who may benefit most from adipokine modulation.

Moreover, while animal models have been instrumental in dissecting the mechanistic roles of adipokines in cardiovascular health, they often fail to capture the full complexity and heterogeneity of human HFpEF. There is a pressing need for more sophisticated models that closely mimic the human disease phenotype, including comorbid conditions, such as obesity, diabetes, and hypertension, that commonly coexist in HFpEF patients. Additionally, longitudinal studies in diverse human populations are essential to validate preclinical findings and to understand the temporal relationship between adipokine dysregulation and HFpEF progression. Such studies should aim to clarify the precise mechanisms by which adipokines contribute to HFpEF and to identify potential therapeutic targets.

Another critical area for future investigation is the development of specific inhibitors or modulators of adipokines. While some progress has been made, as seen with agents, like AdipoRON, targeting adiponectin receptors, more research is needed to develop safe and effective therapies that can modulate adipokine activity. This includes exploring small molecule inhibitors, peptide disruptors, monoclonal antibodies, or other novel modalities that can specifically target leptin, adiponectin, resistin, and other relevant adipokines. Clinical trials assessing the efficacy and safety of these agents in HFpEF patients are crucial.

Furthermore, the interaction of adipokines with other biomarkers and hormonal pathways in HF warrants deeper exploration. The complex relationships between adipokines and natriuretic peptides, inflammatory cytokines, and anabolic hormones, such as testosterone and growth hormone, need to be elucidated. An especially important area of research is the integration of hormonal therapies to manage anabolic hormonal deficiencies prevalent in HF, with potential combination therapies targeting adipokines [101,102,103]. This could lead to synergistic effects in improving cardiac function and patient outcomes.

Additionally, the contradictory findings observed with leptin levels in HFpEF highlight the need for a better understanding of leptin’s cardiovascular functions, the impact of leptin resistance, and its clinical implications. While chronic hyperleptinemia is generally associated with adverse cardiovascular effects, some studies have reported contradictory evidence suggesting potential cardioprotective roles of leptin in specific subgroups. For example, research indicates that, in obese women, higher leptin levels may be associated with lower LV mass and reduced myocardial stiffness, possibly due to leptin’s ability to improve myocardial metabolism and reduce myocardial steatosis. These findings contradict the conventional view and may arise from the complex interplay between leptin resistance and sensitivity, as well as differences based on sex, ethnicity, and obesity status. Understanding these discrepancies is crucial, as they may influence disease progression and response to therapy. Therefore, future research should focus on investigating these contradictory findings, exploring how leptin’s effects differ across diverse populations, and elucidating the underlying mechanisms. This could lead to more personalized treatment approaches and better clinical outcomes for patients with HFpEF.

Finally, future research would greatly benefit from conducting meta-analyses focusing on the relationship between individual adipokines and HFpEF. Such analyses could provide quantitative insights into the magnitude and consistency of the effects of leptin, adiponectin, and resistin on key pathophysiological processes in HFpEF, such as metabolic dysfunction, cardiovascular remodeling, and systemic inflammation. A meta-analysis for each adipokine would help identify potential heterogeneity across studies, elucidate dose–response relationships, and determine the robustness of observed associations. This approach could also aid in clarifying conflicting findings in the literature and contribute to the development of targeted therapeutic strategies for cardiometabolic HFpEF.

Addressing these gaps in evidence is imperative to pave the way for novel diagnostic tools and targeted therapies that harness the full potential of adipokine modulation in HFpEF. By advancing our understanding of the intricate role of adipokines, we can move towards more effective, personalized treatment strategies that ultimately improve patient outcomes and quality of life.

## 6. Conclusions

This comprehensive review has consolidated current understanding and highlighted significant gaps concerning the role of adipokines in the pathophysiology of HFpEF. Adipokines, such as leptin, adiponectin, and resistin, are not merely byproducts of adipose tissue but are central to HFpEF’s pathogenesis, influencing myocardial structure, endothelial function, systemic inflammation, and metabolic regulation. Leptin often exerts detrimental cardiovascular effects by promoting hypertrophy, fibrosis, and endothelial dysfunction. However, contradictory evidence suggests potential cardioprotective roles of leptin in specific subgroups, highlighting the complexity of leptin resistance and sensitivity influenced by factors like sex, ethnicity, and obesity status. Adiponectin, generally considered protective due to its anti-inflammatory and metabolic benefits, presents a paradox in HF. Elevated adiponectin levels in HF patients often correlate with worse outcomes, possibly reflecting adiponectin resistance or compensatory responses to cardiac dysfunction. Resistin contributes to HFpEF through pro-inflammatory and pro-fibrotic actions; although, clinical evidence linking resistin to HFpEF is less definitive. The considerable variability in adipokine responses among individuals emphasizes substantial gaps in understanding their precise roles and interactions. Future research should focus on elucidating the mechanisms underlying adipokine resistance, exploring contradictory findings, and developing targeted therapies that modulate adipokine activity. By advancing our knowledge of these intricate pathways, we can move toward personalized treatment strategies that improve patient outcomes and quality of life in HFpEF.

## Figures and Tables

**Table 1:** Key cardiovascular effects of chronic hyperleptinemia implicated in HFpEF pathophysiology.

Effects	Elaboration	References
Ventricular Hypertrophy	Promotes LV hypertrophy through direct effects on cardiomyocytes and activation of hypertrophic signaling pathways, like JAK/STAT, MAPK, and PI3K/Akt. Additionally, leptin increases ET-1 production, which amplifies pro-hypertrophic signaling and oxidative stress. However, some studies suggest leptin may reduce LV hypertrophy in certain preclinical models.	[16,18,23,24]
Cardiac Remodeling	Induces myocardial fibrosis and structural changes partly through aldosterone-mediated pathways and increased MMP-2 and collagen III production, leading to adverse cardiac remodeling and stiffness. Leptin-induced ET-1 production further contributes by stimulating fibroblast activation and collagen synthesis.	[16,18,23,24]
Diastolic Dysfunction	Results from the combined effects of myocardial hypertrophy, interstitial fibrosis, and impaired calcium handling, affecting relaxation (lusitropy) and compliance.	[16,18,23,24,25]
Increases Central Sympathetic Outflow	Increases SNS activity, elevating heart rate and potentially raising blood pressure and afterload.	[23]
Vascular Remodeling and Arterial Stiffness	Promotes smooth muscle proliferation and collagen deposition in vessel walls, leading to vascular remodeling and increased arterial stiffness. However, some preclinical studies show conflicting results.	[34,35,36,37]
Endothelial Dysfunction	Reduces NO bioavailability and promotes oxidative stress, impairing endothelium-dependent vasodilation and worsening cardiovascular outcomes.	[34,35,36,37]
Low-Grade Inflammation	Activates inflammatory pathways, increasing cytokine levels (e.g., IL-6, TNF-α) and perpetuating systemic inflammation.	[40,41,42]
Leptin Resistance	Leptin resistance impairs its normal metabolic effects, leading to systemic insulin resistance and exacerbating obesity-related metabolic dysfunction. Despite systemic resistance, cardiovascular tissues often retain sensitivity to leptin’s deleterious effects, a phenomenon called selective leptin resistance.	[19,43]
Impaired Exercise Capacity	Leptin-driven mechanisms, including increased sympathetic tone, diastolic dysfunction, and vascular stiffness, contribute to reduced exercise tolerance in HFpEF.	[57]
Potential Protective Effects in Certain Subgroups	Some studies report that higher leptin levels are associated with lower LV mass and reduced myocardial stiffness in specific populations, such as obese African American women, indicating possible cardioprotective effects.	[53,54]

Abbreviations. ET-1 (endothelin-1); HFpEF (heart failure with preserved ejection fraction); IL-6 (interleukin-6); JAK/STAT (Janus kinase/signal transducers and activators of transcription); LV (left ventricular); MAPK (mitogen-activated protein kinase); MMP-2 (matrix metalloproteinase-2); NO (nitric oxide); PI3K/Akt (phosphatidylinositol 3-kinase/Akt pathway); SNS (sympathetic nervous system); TNF-α (tumor necrosis factor-alpha).

**Table 2:** Key cardiovascular effects of adiponectin implicated in HFpEF pathophysiology.

Effects	Elaboration	References
Metabolic Regulation	Activates AMPK signaling to increase glucose uptake and fatty acid oxidation, reducing lipid accumulation and oxidative stress in cardiomyocytes, thereby regulating cardiac metabolism.	[58,59]
Endothelial Function	Improves endothelial function by increasing NO bioavailability through eNOS activation, counteracting endothelial dysfunction and promoting vasodilation. Some studies suggest that these beneficial effects may be attenuated in HF due to adiponectin resistance or may not correlate with improved outcomes in advanced disease stages.	[14,61,62,63,64,65,76,77,78,79]
Anti-Inflammatory Effects	Inhibits NF-κB signaling, reduces pro-inflammatory cytokines (e.g., TNF-α, IL-6), and decreases adhesion molecules, like VCAM-1, limiting inflammatory cell recruitment to the vascular wall. Contradictory findings indicate that, in HF, elevated adiponectin levels may be associated with systemic inflammation and worse clinical outcomes; however, this is possibly just correlation rather than causation.	[14,41,61,62,63,64,65,66,67,70,76,77,78,79]
Anti-Hypertrophic Effects	Prevents cardiomyocyte hypertrophy through AMPK-mediated inhibition of ERK activation. However, elevated adiponectin levels in HF patients have been linked to worse cardiac remodeling, while this is possibly just correlation rather than causation.	[14,66,69,70,71,76,77,78,79]
Anti-Fibrotic Effects	Attenuates angiotensin II-induced cardiac fibrosis via PPAR-α activation, reducing extracellular matrix deposition and myocardial stiffening, improving diastolic function. Yet, in advanced HF, elevated adiponectin levels may reflect a state of adiponectin resistance, limiting these protective effects.	[14,66,69,70,71,76,77,78,79]
Decreases Central Sympathetic Outflow	Decreases SNS activity, potentially lowering blood pressure. However, the impact on sympathetic activity may vary.	[78,79]
Oxidative Stress Reduction	Lowers ROS production and improves calcium handling in cardiomyocytes, enhancing myocardial efficiency and diastolic function. In HF, the relationship between adiponectin levels and oxidative stress is complex, and elevated levels may indicate increased oxidative stress rather than protection.	[14,41,60,68,76,77,78,79]
Improved Exercise Capacity	Adiponectin-driven cardioprotective mechanisms contribute to improved exercise tolerance in HFpEF.	[57]
Adiponectin Paradox in HF	Elevated adiponectin levels in HF may reflect a compensatory maladaptive response or the presence of adiponectin resistance, often associated with worse clinical outcomes, cachexia, and systemic inflammation. This paradox suggests that high adiponectin levels do not always confer cardioprotective effects and may signify disease severity.	[14,76,77,78,79]

Abbreviations. AMPK (AMP-activated protein kinase); eNOS (endothelial nitric oxide synthase); ERK (extracellular signal-regulated kinase); HF (heart failure); HFpEF (heart failure with preserved ejection fraction); IL-6 (interleukin-6); NF-κB (nuclear factor-kappa B); NO (nitric oxide); PPAR-α (peroxisome proliferator-activated receptor alpha); ROS (reactive oxygen species); SNS (sympathetic nervous system); TNF-α (tumor necrosis factor-alpha); VCAM-1 (vascular cell adhesion molecule-1).

**Table 3:** Key cardiovascular effects of resistin implicated in HFpEF pathophysiology.

Effects	Elaboration	References
Metabolic Dysregulation	Inhibits AMPK, reducing glucose uptake and increasing hepatic gluconeogenesis, contributing to hyperglycemia and insulin resistance. Also promotes dyslipidemia by altering lipid metabolism. However, clinical studies show inconsistent associations between resistin levels and insulin resistance, suggesting a complex role in metabolic regulation.	[5,59]
Inflammation	Activates NF-κB, increasing the production of pro-inflammatory cytokines, such as TNF-α, IL-6, and MCP-1, perpetuating a chronic inflammatory state. Upregulates adhesion molecules, like VCAM-1 and ICAM-1, facilitating leukocyte infiltration and exacerbating vascular inflammation, contributing to atherosclerosis. Some studies indicate that resistin’s pro-inflammatory effects may vary between species and tissues, adding complexity to its role in inflammation.	[5,14,41,82,83]
Endothelial Dysfunction	Reduces NO bioavailability by increasing ROS production and elevating ET-1 levels, impairing vasodilation and promoting vascular complications. However, conflicting evidence exists regarding resistin’s direct impact on endothelial function in humans, suggesting that other factors may modulate this effect.	[5,14,41,82,83]
Fibrosis and Cardiac Remodeling	Stimulates collagen synthesis in cardiac fibroblasts via pro-fibrotic pathways, like TGF-β/Smad, increasing myocardial stiffness and promoting diastolic dysfunction. Yet, clinical studies have not consistently linked resistin levels with myocardial fibrosis in HFpEF patients, indicating potential differences between experimental models and human disease.	[84,85,86,87,88]
Calcium Handling and Contractility	Disrupts calcium homeostasis in cardiomyocytes by impairing SERCA2a function and increasing calcium leak, reducing diastolic relaxation (lusitropy) and myocardial contractility. Limited clinical data are available to confirm these mechanisms in HFpEF patients, highlighting a gap between experimental findings and clinical observations.	[87,89,90,91]
Oxidative Stress	Upregulates NADPH oxidase, increasing ROS production and causing mitochondrial dysfunction. This accelerates cardiomyocyte apoptosis and exacerbates cardiac tissue damage. However, the extent to which resistin contributes to oxidative stress in human HFpEF remains unclear due to inconsistent clinical evidence.	[5,14,41,82,83]
Neurohormonal Activation	Enhances sympathetic nervous system activity, elevating blood pressure and heart rate, which increases myocardial afterload. Also modulates RAAS, contributing to hypertension and HF progression. Contradictory findings suggest that resistin’s role in neurohormonal activation may not be as significant in humans as observed in animal models.	[91,92,93]
Impaired Exercise Capacity	Resistin-driven mechanisms, including increased sympathetic tone, diastolic dysfunction, and vascular stiffness, contribute to reduced exercise tolerance in HFpEF.	[57]
Limited Association with HFpEF in Humans	Clinical studies have not consistently demonstrated a significant association between resistin levels and HFpEF incidence or progression. Some studies find a stronger link with HFrEF, suggesting that resistin’s role in HF may be subtype-specific or influenced by other comorbidities.	[97]

Abbreviations. AMPK (AMP-activated protein kinase); HF (heart failure); HFrEF (heart failure with reduced ejection fraction); HFpEF (heart failure with preserved ejection fraction); ICAM-1 (intercellular adhesion molecule-1); IL-6 (interleukin-6); MCP-1 (monocyte chemoattractant protein-1); NADPH (nicotinamide adenine dinucleotide phosphate); NF-κB (nuclear factor-kappa B); NO (nitric oxide); RAAS (renin–angiotensin–aldosterone system); ROS (reactive oxygen species); SERCA2a (sarcoplasmic/endoplasmic reticulum calcium ATPase 2a); SNS (sympathetic nervous system); TGF-β (transforming growth factor-beta); TNF-α (tumor necrosis factor-alpha); VCAM-1 (vascular cell adhesion molecule-1).

## Data Availability

Data sharing is not applicable.
